# Repurposing an Antioxidant to Kill *Mycobacterium tuberculosis* by Targeting the 50S Subunit of the Ribosome

**DOI:** 10.3390/biom13121793

**Published:** 2023-12-14

**Authors:** Wenqi Dong, Gaoyan Wang, Yajuan Bai, Yuxin Li, Liying Zhao, Wenjia Lu, Chenchen Wang, Zhaoran Zhang, Hao Lu, Xiangru Wang, Huanchun Chen, Chen Tan

**Affiliations:** 1 National Key Laboratory of Agricultural Microbiology, College of Veterinary Medicine, Huazhong Agricultural University, Wuhan 430070, China; dongwq@mail.hzau.edu.cn (W.D.); wgy_1993hzau@163.com (G.W.); baiyajuan@webmail.hzau.edu.cn (Y.B.); liyuxin@webmail.hzau.edu.cn (Y.L.); zly2002@webmail.hzau.edu.cn (L.Z.); 2017302110131@webmail.hzau.edu.cn (W.L.); 2018302110164@webmail.hzau.edu.cn (C.W.); zhangzhaoran@mail.hzau.edu.cn (Z.Z.); sdluhao521@163.com (H.L.); wangxr228@mail.hzau.edu.cn (X.W.); chenhch@mail.hzau.edu.cn (H.C.); 2 The Cooperative Innovation Center for Sustainable Pig Production, Wuhan 430070, China; 3 Key Laboratory of Preventive Veterinary Medicine in Hubei Province, Wuhan 430070, China; 4 Hubei Hongshan Laboratory, Wuhan 430070, China

**Keywords:** *Mycobacterium tuberculosis*, SKQ-1, anti-tuberculosis, 50S ribosomal subunit, protein translation

## Abstract

Tuberculosis and drug-resistant TB remain serious threats to global public health. It is urgent to develop novel anti-TB drugs in order to control it. In addition to redesigning and developing new anti-TB drugs, drug repurposing is also an innovative way to develop antibacterial drugs. Based on this method, we discovered SKQ-1 in the FDA-approved drug library and evaluated its anti-TB activity. In vitro, we demonstrated that SKQ-1 engaged in bactericidal activity against drug-sensitive and -resistant Mtb and confirmed the synergistic effects of SKQ1 with RIF and INH. Moreover, SKQ-1 showed a significant Mtb-killing effect in macrophages. In vivo, both the SKQ-1 treatment alone and the treatment in combination with RIF were able to significantly reduce the bacterial load and improve the survival rate of *G. mellonella* infected with Mtb. We performed whole-genome sequencing on screened SKQ-1-resistant strains and found that the SNP sites were concentrated in the 50S ribosomal subunit of Mtb. Furthermore, we proved that SKQ-1 can inhibit protein translation. In summary, from the perspective of drug repurposing, we discovered and determined the anti-tuberculosis effect of SKQ-1, revealed its synergistic effects with RIF and INH, and demonstrated its mechanism of action through targeting ribosomes and disrupting protein synthesis, thus making it a potential treatment option for DR-TB.

## 1. Introduction

Tuberculosis (TB) causes an estimated 1.4 million deaths annually [1]. The emergence of drug-resistant tuberculosis (DR-TB) has increased the difficulty of controlling and eliminating TB. It is urgently necessary to develop and find new drugs for TB treatment. The development of new drugs requires sufficient time for clinical trials. Therefore, in recent years, old drugs have been reused to find anti-TB drugs [2,3,4,5]. The anti-TB effects of linezolid, clofazimine, and meropenem were discovered through repurposing old drugs [6]. Nonsteroidal anti-inflammatory drugs (NSAIDs) have attracted attention in the repurposing of anti-TB drugs due to their versatility and low cost. Hydroxyphenylbutazone demonstrated antibacterial activity against replicating, dormant, and drug-resistant *Mycobacterium tuberculosis* (Mtb) [7]. Aspirin and ibuprofen synergized with the first-line anti-tuberculosis drug pyrazinamide to significantly reduce the tissue bacterial burden in a mouse model infected with Mtb [8,9]. The non-antibiotic metformin, which is derived from leguminous plants and is mainly used in the treatment of type 2 diabetes, was revealed to have a characteristic anti-tuberculosis activity that can reduce the risk of developing active TB [10,11]. We identified the antimycobacterial activity of SKQ-1, a mitochondria-targeted antioxidant, which can reduce the production of mitochondrial reactive oxygen species [12,13]. It can also be used to treat dry eye and light-induced retinal degeneration [14,15,16,17]. However, its antimycobacterial activity has not been reported.

Here, we report the in vitro antibacterial activity of SKQ-1 against Mtb H37Rv and clinically isolated drug-resistant Mtb. We explored the effects of SKQ-1 in combination with the first-line antimycobacterial drug RIF in vivo and in vitro. We revealed the antibacterial mechanism of SKQ-1 through sequencing the genome of SKQ-1-drug-resistant strains and detecting protein transcription/translation efficiency.

## 2. Materials and Methods

### 2.1. Drugs and Reagents

SKQ-1 and bedaquiline (BDQ) were obtained from TargetMol (Shanghai, China). Isoniazid (INH), ethambutol (EMB), LNZ (linezolid), and rifampicin (RIF) were obtained from Selleck (Shanghai, China). Dulbecco’s modified Eagle’s (DMEM) medium and fetal bovine serum (FBS) were purchased from Gibco (New York, NY, USA). Middlebrook 7H9 medium, Middlebrook 7H11 medium 7H9, and oleic acid, albumin, and dextrose (OADC) were purchased from Becton Dickinson (Franklin Lakes, NJ, USA). Glycerol, Tween 80, and dimethyl sulfoxide (DMSO) were purchased from Sigma-Aldrich (St. Louis, MO, USA). A WST-1 cell proliferation and cytotoxicity assay kit and an *E. coli* S30 circular DNA assay kit (L1020) were purchased from Beyotime (Shanghai, China) and Promega (Wisconsin, DC, USA), respectively.

Glycerol, Tween 80, and dimethyl sulfoxide (DMSO), Dulbecco’s modified Eagle’s (DMEM) medium and fetal bovine serum (FBS), and a WST-1 cell proliferation and cytotoxicity detection kit and an *E. coli* S30 circular DNA detection kit (L1020) were purchased from Sigma-Aldrich, Gibco, Biyuntian, and Promega (Madison, WI, USA), respectively.

### 2.2. M. tuberculosis Strains and Cell Culture

DR-Mtb strains were donated by Heze Fifth People’s Hospital. The reference strain H37Rv of Mtb (ATCC 27294) and the isolated DR-Mtb strains were resuscitated on solid Middlebrook 7H11 medium (10% OADC and 0.5% glycerol) and cultured in liquid Middlebrook 7H9 medium (10% OADC, 0.5% glycerol, and 0.05% Tween 80). Experiments related to Mtb were all carried out in a Biosafety Level 3 facility at Huazhong Agricultural University (Wuhan, China). THP-1 human monocyte-macrophage cells and Vero cells were cultured in RPMI-1640 medium and DMEM medium supplemented with 10% FBS, respectively, and they were cultured at 37 °C with 5% CO_2_.

### 2.3. MIC Determination

The minimum inhibitory concentration (MIC) was determined using a resazurin-reducing microplate assay (REMA) [18]. Drugs were serially diluted twofold in 96-well plates supplemented with 100 μL of 7H9 medium. A negative control without a drug and a positive control with RIF were set up at the same time. Then, 1 × 10^5^ logarithmic-phase bacteria were added to each well and cultured at 37 °C in an incubator for 7 days. Subsequently, 30 μL of 0.01% resazurin was added to each well of plates, followed by incubation at 37 °C for 24 h. A change from blue to pink indicates bacterial growth. Therefore, the MIC was defined as the lowest drug concentration that prevented color change.

### 2.4. Cytotoxicity Determination

A total of 1 × 10^4^ Vero cells were seeded into 96-well cell plates and cultured in DMEM (10% FBS) containing different concentrations of SKQ-1 at 37 °C with 5% CO_2_. Vero cells were cultured for 24 h and another 2 h after adding 10 μL of WST-1 to each well. The 96-well cell plate was shaken for 1 min before the measurement of the fluorescence value with a fluorescence microplate reader (TECAN SPARK 10M, TECAN, Grödig, Austria) at OD450. The minimum concentration that inhibited at least 50% of cell growth was determined and defined as IC50.

### 2.5. Kill Curve Assay

An analysis of the bactericidal kinetics was carried out according to a previous description [18]. We diluted the log-phase H37Rv to an OD600 of 0.05 and cultured the bacteria at final concentrations of 1 μg/mL, 4 μg/mL, 8 μg/mL, and 16 μg/mL SKQ-1 in 7H9 medium. The log-phase bacteria cultured without a drug and those with a final concentration of 0.25 μg/mL INH were used as the negative control and positive control, respectively. The cultures were serially diluted tenfold and plated on 7H11 agar medium at 0 days, 2 days, 4 days, 8 days, and 12 days post-incubation. The plates were cultured at 37 °C for 4 weeks, and the colonies were counted.

### 2.6. Drug Synergy according to the Checkerboard Method

The combined effects of SKQ-1 and anti-tuberculosis drugs, including RIF, INH, EMB, LNZ, and BDQ, on M.tb H37Rv were determined using the checkerboard method [18]. Serial twofold dilutions of SKQ-1 and one anti-tuberculosis drug were added to 96-well plates, and then 50 μL of logarithmic-phase Mtb diluted to an OD600 of 0.05 was added. The determination of MIC was carried out according to the above-described method. The fractional inhibitory concentration index (FICI) and the total fractional inhibitory concentration (FIC) of the two drugs were used to indicate the combined effects of the two drugs. The FIC was calculated with the following formula: FIC = MIC of the drug used alone/MIC of the drug used in combination. FICI ≤ 0.5, FICI = 0.5–4.0, and FICI ≥ 4.0, respectively, indicated that the combination of the two drugs had a synergistic effect, an additive effect, or different antagonistic effects [19].

### 2.7. Intracellular Antibacterial Activity Assay

A total of 4 × 10^5^ THP-1 cells were seeded per well in 24-well plates and induced for 48 h with complete RPMI-1640 medium at a final concentration of 100 ng/mL PMA so that they would differentiate into macrophages. Subsequently, the macrophages were cultured in RPMI-1640 medium for 12 h and then infected with log-phase H37Rv at an MOI of 5 for 4 h [20]. Cells were washed 3 times with PBS to remove extracellular bacteria and were treated with RPMI-1640 medium (2% FBS) containing different final concentrations of the drugs until 48 h after infection. Macrophages were lysed with 0.025% Triton X-100, and the lysates were diluted into an appropriate gradient in PBS, plated on 7H11 agar medium, and incubated at 37 °C for intracellular bacterial survival assays.

### 2.8. Protective Effect of SKQ-1 on Galleria mellonella In Vivo

Due to the limitations of the conditions of the Animal Biosafety Level 3 laboratory, we chose the *Galleria mellonella* (*G. mellonella*) model to evaluate the in vivo protective effect of SKQ-1. To determine the appropriate amount of H37Rv in the *G. mellonella* infection model, we used various amounts of bacteria to infect *G. mellonella* and observe its survival. The minimum infectious dose that killed all *G. mellonella* within three days was determined as the infectious dose for the subsequent experiments.

A total of 45 *G. mellonella* with good growth and flexible movement were selected and injected with the minimum lethal dose of H37Rv; then, they were randomly divided into three groups, with 15 *G. mellonella* in each group. At 24 h post-infection, the *G. mellonella* were separately treated with different drugs. The RIF group and the SKQ-1 group were treated with 10 mg/kg of RIF and 10 mg/kg of SKQ-1, respectively. The SKQ-1+RIF group involved a combination of 10 mg/kg of RIF and 10 mg/kg of SKQ-1. The *G. mellonella* were cultured at 37 °C, and their survival was continuously observed [21]. We fixed them in 4% paraformaldehyde before death or 5 days post-treatment and embedded them in paraffin. The embedded *G. mellonella* were cut into sections that were 2–3 μm thick. We observed the *M. tuberculosis* burden in *G. mellonella* using light microscopy (OLYMPUS, BX41-12P02 Tokyo, Japan) on acid-fast stained sections [22].

### 2.9. Mutation Frequency and Acquisition of Resistant Mutants

A total of 1 × 10^9^ log-phase H37Rv cells were seeded on 7H11 plates at concentrations of 8×, 16×, and 32× the MIC, respectively. Single colonies were picked from plates incubated at 37 °C for 1 month and inoculated into 7H9 medium containing SKQ-1. The genomes of the resistant mutant strains and the wild-type laboratory strain were extracted and subjected to whole-genome sequencing. The sequencing data were obtained based on the Illumina sequencing platform, and variant detection was performed through alignment with the H37Rv reference genome. The genomic DNA underwent quality inspection, library construction, detection, and sequencing to obtain clean data. The clean data were aligned with the Mtb reference genome (H37Rv NC_000962.3) to obtain the sequencing depth and coverage statistics (BWA 0.7.17) [23].

### 2.10. The Coupled Transcription/Translation Assay

An *E. coli* S30 circular DNA assay kit was used to detect the inhibition of compounds in bacterial translation. The 50 µL reaction mixture contained 20 µL of S30 premix without amino acids, 15 µL of S30 extract, 5 µL of a complete amino acid mixture, 2 µg of pBESTluc™ DNA, nuclease-free water, and the detected drug (1 μg/mL, 2 μg/mL, 4 μg/mL and 8 μg/mL SKQ-1; 0.5 μg/mL LNZ was used as a positive control, and DMSO was used as a negative control). After gentle vortexing and brief centrifugation, the mixture was incubated at 37 °C for 1 h, followed by an ice bath for 5 min to terminate the reaction. Then, 50 µL of the luciferase assay reagent was diluted fivefold in luciferase dilution reagent and added to a 96-well black plate, and the fluorescence value was measured after adding the previous reaction systems [24].

### 2.11. Statistical Analysis

We used GraphPad Prism 8.0 for statistical analysis and defined *p* < 0.05 as *, *p* < 0.01 as **, *p* < 0.005 as ***, and ns as not significant. The data were assessed using one-way ANOVA with the Dunnet correction for multiple comparisons.

## 3. Results

### 3.1. Activity of SKQ-1 against Mtb

We preliminarily determined the antibacterial effect of SKQ-1 on the BCG strain using the method of whole-cell screening. Subsequently, we used the resazurin detection method to detect the 90% MICs of SKQ-1 against Mtb H37Rv, the attenuated strain H37Ra, the virulent strain M. bovis, and the vaccine strain BCG. The results showed that the MICs for both H37Rv and H37Ra were 1 μg/mL, and the MICs for M. bovis and BCG were both 2 μg/mL. We examined the antimicrobial activity of SKQ-1 against drug-resistant strains, where the MICs for mono-drug-resistant strains against first-line anti-tuberculosis drugs (RIF, INH, and EMB at 1–2 µg/mL) and the MICs of SKQ-1 against the multidrug-resistant strain at 4 μg/mL (Table 1), proved the strong antibacterial effect on drug-resistant strains. The cytotoxicity of SKQ-1 in Vero cells was determined using the WST-1, and the results showed that the IC50 of SKQ-1 in Vero cells was 32 µg/mL.

### 3.2. Kill Curve Dynamics

**Figure 1:**
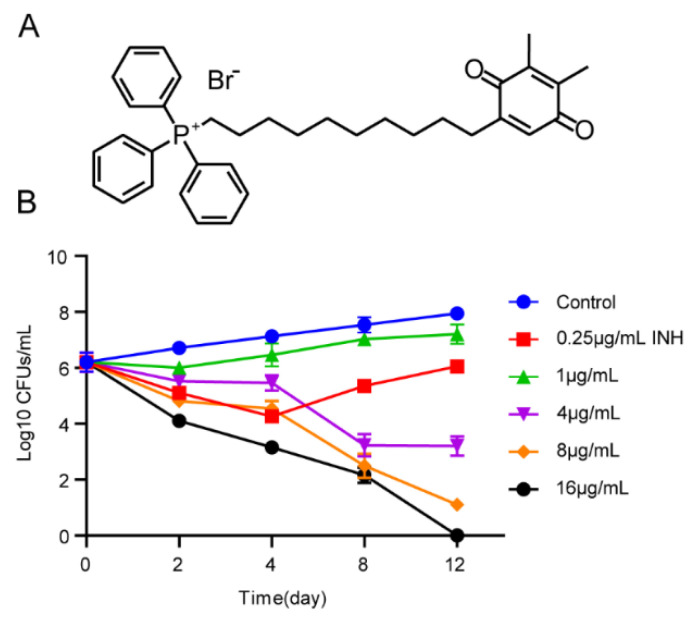
The killing kinetics of SKQ-1 against Mtb. (**A**) Structures of SKQ-1. (**B**) Mtb was treated with 1 μg/mL, 4 μg/mL, 8 μg/mL, and 16 μg/mL SKQ-1, and 0.25 μg/mL. INH was used as a positive control. The CFU was measured at different time points after treatment. Experiments were performed in triplicate, and at least two biological replicates were included. Data are shown as mean ± SD.

To determine the killing kinetics of SKQ-1 against Mtb H37Rv, we performed a CFU analysis of bacteria that were cultured in 7H9 medium at concentrations of 1 μg/mL, 4 μg/mL, 8 μg/mL, and 16 μg/mL SKQ-1 for 0, 2, 4, 8, and 12 days. The results showed that SKQ-1 had a concentration-dependent killing effect on Mtb. The bacteria were reduced by 1 log10 CFU/mL and 4 log10 CFU/mL at 2 days and 12 days, respectively, after treatment with 8 μg/mL SKQ-1. At a concentration of 16 μg/mL, SKQ-1 killed all bacteria on the 12th day (Figure 1). These results indicated that the compound had a significant bactericidal effect on Mtb.

### 3.3. Activity of SKQ-1 against Intracellular Mtb

**Figure 2:**
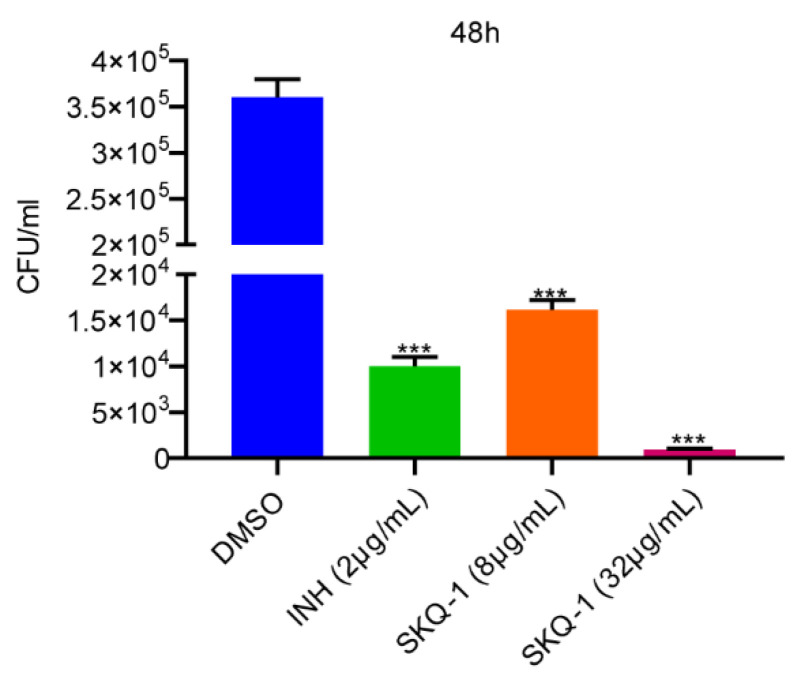
Intracellular bactericidal activity of SKQ-1 against Mtb. THP-1 macrophages were infected with Mtb and treated with SKQ-1 at final concentrations of 8× and 32× the minimum inhibitory concentration (MIC). Macrophages treated with DMSO were used as a negative control. Macrophages treated with isoniazid (INH) at 32× the MIC were used as positive controls. The CFU was measured after 2 days of treatment and recalculated as CFU/mL. Experiments were performed in triplicate, and at least two biological replicates were included. Data were shown as mean ± SD and unpaired Student’s *t*-test or one-way ANOVA was used to conduct statistical significance analysis. (***, *p* < 0.001).

The effect of SKQ-1 on intracellular Mtb was examined through the THP-1 macrophage infection model. Macrophages were incubated with RPMI-1640 medium supplemented with 2% FBS containing 8 μg/mL or 32 μg/mL SKQ-1 until 48 h post-infection. A group cultured with a medium supplemented with DMSO served as a negative control, and a group cultured with a medium with a final concentration of 2 μg/mL INH served as a positive control. At 48 h post-infection, the cells were lysed, and CFU assays were performed to determine the number of viable intracellular bacteria. The bactericidal effect of INH on intracellular H37Rv was consistent with that in previous reports. The effects of 8 μg/mL SKQ-1 and 2 μg/mL INH were similar, and the clearance of H37Rv in macrophages was enhanced with the increase in SKQ-1 concentration. Compared with the negative control group, the group of SKQ-1 at 32 μg/mL exhibited a reduction in intracellular bacteria by 360 times (Figure 2). The above results indicate that SKQ-1 can kill H37Rv in macrophages.

### 3.4. The Antibacterial Synergy of SKQ-1

The TB treatments are long, and long-term use of a single drug would accelerate the development of drug-resistant Mtb strains. The development of effective combination therapy regimens is crucial in reducing the emergence of drug-resistant strains and shortening the duration of treatments. We determined that the MICs of RIF, INH, EMB, LNZ, and BDQ against the H37Rv strain were 0.0156 μg/mL, 0.0625 μg/mL, 1 μg/mL, 0.5 μg/mL, and 0.0625 μg/mL, respectively. Subsequently, we explored the effects of combining SKQ-1 with RIF, INH, EMB, LNZ, and BDQ using the checkerboard method. The results in terms of drug synergy showed that the synergistic effects of SKQ-1 combined with RIF and INH on Mtb H37Rv and the FICIs were 0.1875 and 0.0626, respectively. In combination with SKQ-1, the MICs of RIF and INH in H37Rv decreased from 0.0156 and 0.0625 μg/mL to 0.002 μg/mL; thus, they decreased by 7.8 and 31.25 times, respectively. In addition, there were no synergistic effects of SKQ-1 when combined with EMB, LNZ, or BDQ in Mtb H37Rv, but there was an additive effect; the FICIs were 1.004, 1.0156, and 0.5156, respectively. Furthermore, in combination with SKQ-1, the MIC of BDQ in H37Rv decreased from 0.0625 μg/mL to 0.0312 μg/mL, a twofold decrease (Table 2).

### 3.5. SKQ-1 in Combination with RIF Protects G. mellonella from Mtb

**Figure 3:**
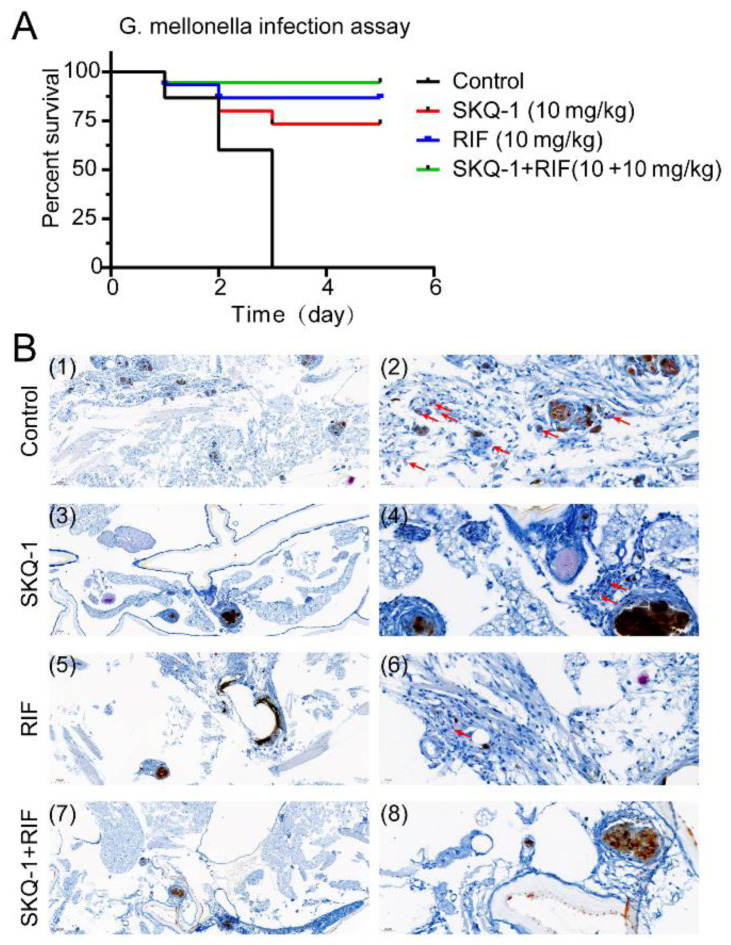
Combined effects of SKQ-1 and rifampicin (RIF) in a *G. mellonella* larvae infection assay. *G. mellonella* larvae (*n* = 15 per group) were infected with H37Rv (5 × 10^6^ CFU). *G. mellonella* larvae were then treated with SKQ-1 (10 mg/kg) or RIF (10 mg/kg) alone or in combination (10 + 10 mg/kg). (**A**) Survival rates of the *G. mellonella* larvae. (**B**) Acid-fast staining of H37Rv in the *G. mellonella* larvae. The scale bars in images (1), (3), (5), and (7) represent 100 μm; the scale bars in images (2), (4), (6), and (8) represent 20 µm. Red arrow points to bacteria.

We used different doses to infect *G. mellonella*; the lowest dose of 5 × 10^6^, which killed all the *G. mellonella* within 3 days, was set as the infectious dose for the subsequent protective experiments. The anti-tubercular effects of SKQ-1 and SKQ-1 combined with RIF were evaluated through observing the survival rate of *G. mellonella* and the results of acid-fast staining. In the control group, the *G. mellonella* without any drug treatment, all died at 3 days post-infection, and multiple aggregates of Mtb were observed using acid-fast staining. The *G. mellonella* survival rates in the SKQ-1 and RIF groups were 75% and 80%, respectively. Compared with those in the control group, Mtb aggregates were significantly reduced in the single-drug treatment groups in vivo. The combination of SKQ-1 and RIF increased the survival rate of *G. mellonella* to 90% (Figure 3), and Mtb was not observed in this group (Figure 3). The above results indicate that SKQ-1 is active against Mtb in vivo, and its use in combination with RIF can enhance its therapeutic effect.

### 3.6. SKQ-1 Targets Ribosomes in Mtb

To reveal the mutation frequency, we collected 1 × 10^9^ log-phase H37Rv cells and coated them on 8×, 16×, and 32× MIC plates; then, they were cultured at 37 °C for one month. We obtained two single colonies on the 32× MIC plate, but no colonies were observed on the 8× and 16× MIC plates. We calculated the resistance frequency of Mtb to SKQ-1 to be 0.5 × 10^−8^/CFU from the initial inoculation and the number of mutant colonies obtained. Previous studies indicated that the mutation frequencies of INH and RIF were 10^−6^ and 10^−7^ to 10^−8^, respectively [25]. The results indicated that SKQ-1 had a lower mutation frequency during use.

To explore the target of SKQ-1, we performed whole-genome sequencing of two SKQ-1-resistant strains and a wild-type laboratory strain. Through aligning the results with the sequence of the reference genome, we found that the genomic sequence changes that were present in both the mutant strains but not in the wild-type laboratory strain were located on the 50S subunits of Mtb. There were 14 single-nucleotide polymorphisms (SNPs) localized in the 50S ribosome (Table 3), suggesting that SKQ-1 targets the ribosome in Mtb. The normal function of the ribosome is an indispensable prerequisite for ensuring protein synthesis in Mtb. Linezolid, which is an oxazolidinone, kills Mtb through interacting with the 23S rRNA of the 50S ribosome and interfering with protein synthesis [26,27].

**Figure 4:**
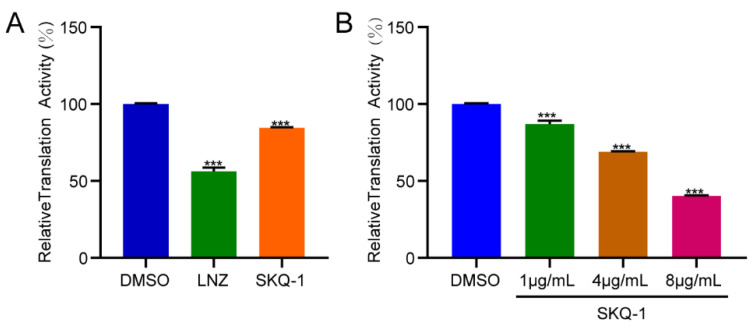
The inhibition of translation by SKQ-1. The 50 µL reaction systems contained S30 premix, S30 extract, a complete amino acid mixture, pBESTluc™ DNA, and different concentrations of drugs; these were used to detect the inhibition of protein synthesis. (**A**) The relative translation activity of linezolid (LNZ) at 0.5 μg/mL and SKQ-1 at 2 μg/mL. (**B**) The relative translation activity of SKQ-1 at 1 μg/mL, 4 μg/mL, and 8 μg/mL. Experiments were performed in triplicate, and at least two biological replicates were included. Data were shown as mean ± SD and unpaired Student’s *t*-test or one-way ANOVA was used to conduct statistical significance analysis. (***, *p* < 0.001).

To confirm the effect of SKQ-1 on protein synthesis, we performed a coupled transcription/translation analysis. DMSO and 0.5 μg/mL LNZ were added to the reaction systems as a negative control and positive control, respectively. We detected the inhibitory effect of 2 μg/mL SKQ-1 on translation. The results showed that LNZ significantly inhibits protein translation, which was consistent with the findings of previous studies, and SKQ-1 exhibited the same effect as that of LNZ (Figure 4A). Subsequently, we measured the inhibition of translation after treatment with different concentrations of SKQ-1 (1 μg/mL, 4 μg/mL, and 8 μg/mL), and the results showed that the inhibition of protein synthesis was enhanced as the concentration increased. It was also found that 8 μg/mL SKQ-1 was able to reduce the translation activity to 40% (Figure 4B).

## 4. Discussion

The development of new drugs for TB therapy requires high costs and a long time, but repurposing previously discovered TB drugs is faster and more effective. Drugs that have been approved for use have relatively complete safety data and clinical experience. SKQ-1 is an approved drug for the treatment of dry eye syndrome in the FDA Drug Library. The relatively low cytotoxicity of SKQ-1 in cells is a prerequisite for TB treatment.

The survival of Mtb in host macrophages is why it is difficult to clear it. The ability to kill intracellular Mtb is also an important indicator for evaluating anti-tuberculosis drugs. We found that SKQ-1 showed a significant killing ability against Mtb in macrophages. Thus, SKQ-1 has the potential to become a clinical treatment for tuberculosis.

INH, RIF, and EMB play a vital role in the clinical treatment of TB [28]. BDQ is a selective Mtb ATP synthase inhibitor; it is active against replicating and dormant Mtb and can be used to treat MDR-TB [29,30]. The oxazolidinone antibiotic linezolid, which inhibits protein synthesis via binding to the bacterial 50S ribosomal subunit, was discovered through drug repurposing and is recommended by the WHO for the treatment of patients infected with MDR-TB or XDR-TB infection [31,32]. We measured the combined effects of SKQ-1 and these anti-tuberculosis drugs. The results showed that SKQ-1 had a synergistic effect when used in combination with RIF and INH, and it had an additive effect when used with EMB, BDQ, and LNZ. We identified the single-nucleotide mutations of SKQ-1-resistant strains located in the 23S rRNA, which is an important component of the ribosomal 50S subunit, and we demonstrated that SKQ-1 impairs protein synthesis through inhibiting translation. Since both SKQ-1 and LNZ target the 50S subunit, there were no synergistic effects when the two drugs were used in combination. SKQ-1 synergizes with RIF, which targets RNA polymerase to repress Mtb transcription [33], thus impairing protein synthesis. It also had good synergistic effects both in vivo and in vitro. We detected the MIC of SKQ-1 against clinically isolated resistant strains. The results showed that the MICs of RIF, INH, and EMB single-drug-resistant tuberculosis were 1–2 μg/mL, and the MICs for the two MDR-TB strains were 4 and 8 μg/mL, respectively.

In summary, we identified an FDA-approved drug, SKQ-1, which targets the 50S subunit of the ribosome of Mtb to inhibit protein synthesis. We demonstrated the effect of SKQ-1 on clinically isolated drug-resistant strains and the synergistic effect of SKQ-1 and RIF in vitro and in vivo. It is suggested that SKQ-1 can be a candidate drug for drug-susceptible tuberculosis and mono-drug-resistant tuberculosis treatment.

## Figures and Tables

**Table 1:** MICs of SKQ-1 against strains of *M. tuberculosis*.

Strains	MIC (μg/mL)
H37Rv (Susceptible)	1
*M. bovis* (Susceptible)	2
H37Ra (Susceptible)	1
BCG (Susceptible)	2
CR1 (RIF-R)	2
CR2 (RIF-R)	2
CR3 (INH-R)	2
CR4 (INH-R)	1
CR5 (EMB-R)	1
CR6 (MDR)	4

MIC: the minimum inhibitory concentration; *M. bovis*: *Mycobacterium bovis*; RIF: rifampicin; INH: isoniazid; EMB: ethambutol.

**Table 2:** Effects of Combining SKQ-1 with RIF, INH, EMB, LNZ, and BDQ to treat Mtb.

Drug	MIC (μg/mL)		FIC	FICI	Remarks
Combination	Alone	Combination			
SKQ-1	1	0.0625	0.0625	0.1875	synergy
RIF	0.0156	0.002	0.125		
SKQ-1	1	0.0312	0.0312	0.0624	synergy
INH	0.0625	0.002	0.0312		
SKQ-1	1	0.004	0.004	1.004	additive
EMB	1	1	1		
SKQ-1	1	0.0156	0.0156	1.0156	additive
LNZ	0.5	0.5	1		
SKQ-1	1	0.0156	0.0156	0.5156	additive
BDQ	0.0625	0.0312	0.5		

LNZ: linezolid; BDQ: bedaquiline.

**Table 3:** Single-nucleotide polymorphisms were detected in the two SKQ-1-resistant Mtb strains.

Type	Location	Reference	After	Gene	Annotation
SNP	1476234	G	A	*rrl*	23S ribosomal RNA
SNP	1476260	A	G	*rrl*	23S ribosomal RNA
SNP	1476268	A	T	*rrl*	23S ribosomal RNA
SNP	1476332	G	C	*rrl*	23S ribosomal RNA
SNP	1476353	G	T	*rrl*	23S ribosomal RNA
SNP	1476358	T	C	*rrl*	23S ribosomal RNA
SNP	1476408	G	A	*rrl*	23S ribosomal RNA
SNP	1476466	C	T	*rrl*	23S ribosomal RNA
SNP	1476481	T	C	*rrl*	23S ribosomal RNA
SNP	1476506	T	C	*rrl*	23S ribosomal RNA
SNP	1476530	C	T	*rrl*	23S ribosomal RNA
SNP	1476547	C	T	*rrl*	23S ribosomal RNA
SNP	1476567	C	T	*rrl*	23S ribosomal RNA
SNP	1476584	C	T	*rrl*	23S ribosomal RNA

## Data Availability

The data are not publicly available due to privacy.
